# UBE2O Drives Immune Evasion and Radioimmunotherapy Resistance in Lung Cancer by Degrading CDKL1 to Induce PD‐L1 Transcription

**DOI:** 10.1002/advs.76223

**Published:** 2026-06-22

**Authors:** Huichan Xue, Xiaohua Jie, Zhiwei Liu, Ruoxin Fang, Shuo Wu, Xiaorong Dong, Shuangbing Xu

**Affiliations:** ^1^ Cancer Center, Hubei Key Laboratory of Precision Radiation Oncology, Institute of Radiation Oncology, Union Hospital, Tongji Medical College Huazhong University of Science and Technology Wuhan Hubei China; ^2^ Key Laboratory of Biological Targeted Therapy (Huazhong University of Science and Technology), Ministry of Education Wuhan Hubei China

**Keywords:** immunotherapy, lung cancer, PD‐L1, radiotherapy, UBE2O

## Abstract

Resistance to radioimmunotherapy is one of the primary causes of treatment failure in lung cancer patients; however, the underlying mechanisms remain inadequately understood. In this study, we found that the E2/E3 hybrid ubiquitin enzyme UBE2O interacted with CDKL1, facilitating its ubiquitination and subsequent degradation at the K21 residue and thereby negatively regulating the stability of the CDKL1 protein in lung cancer. Silencing UBE2O stabilized CDKL1 to inhibit YBX1‐mediated PD‐L1 transcription, which consequently promoted the infiltration and activation of CD8^+^ T cells, thereby enhancing the antitumor immune response in lung cancer. Furthermore, targeting UBE2O in combination with radiotherapy and anti‐PD‐L1 immunotherapy resulted in stronger antitumor efficacy in lung cancer models. Notably, the UBE2O crosslinking inhibitor arsenic trioxide (ATO) reduced PD‐L1 expression and enhanced the efficacy of radioimmunotherapy in preclinical lung cancer models while exhibiting acceptable short‐term tolerability. Our findings indicate that targeting the UBE2O/CDKL1 axis may represent a highly promising strategy for increasing lung cancer sensitization to radioimmunotherapy.

## Graphical abstract

UBE2O targets CDKL1 for the degradation to upregulate PD‐L1 expression and promotes resistance to radioimmunotherapy in lung cancer, supporting the potential of targeting UBE2O as a promising therapeutic strategy.

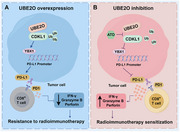

## 1 Introduction

Lung cancer is the malignant neoplasm with the highest incidence and mortality rates worldwide [1, 2]. Radiotherapy combined with immunotherapy has emerged as a primary treatment modality for lung cancer patients [3]. However, treatment failure ultimately occurs in lung cancer patients because of the therapeutic resistance [4]. Consequently, elucidating the mechanisms underlying radioimmunotherapy resistance and identifying novel therapeutic targets have significant theoretical and clinical implications for lung cancer treatment.

Immune checkpoint inhibitors, which are representative drugs for tumor immunotherapy, have demonstrated significant success in the treatment of malignant tumors [5, 6]. Studies have shown that the binding of PD‐L1 on tumor cells to PD‐1 on T cells triggers downstream signaling pathways, leading to the suppression of T cell proliferation and the production and release of cytokines, which ultimately reduces cytotoxic effector functions and compromises immune surveillance against tumors [7]. Nevertheless, the clinical efficacy of immune monotherapy remains limited, and combination strategies with other therapeutic modalities may yield superior treatment outcomes [8]. Emerging evidence indicates that the combination of PD‐1/PD‐L1 inhibition and radiotherapy may exert synergistic antitumor effects [9, 10]. Furthermore, the combination of anti‐TGF‐β/PD‐L1 bispecific antibodies and radiotherapy not only augments antitumor immunity but also has the potential to mitigate radiation‐induced pulmonary fibrosis [11]. Elevated PD‐L1 levels contribute to the persistent activation of STING signaling in tumor cells, leading to increased expression of IFN‐β and IRDS, thereby increasing cancer cell resistance to radiation [12]. Therefore, the identification of novel molecules that regulate PD‐L1 expression and function is essential for elucidating the resistance of lung cancer cells to radioimmunotherapy.

The ubiquitin‐proteasome system (UPS) is the principal system responsible for intracellular protein degradation in eukaryotic cells [13, 14, 15]. The UPS governs the ubiquitination and subsequent degradation of more than 80% of intracellular proteins, highlighting its essential role in maintaining protein homeostasis [15]. The system comprises three main components: a ubiquitin‐activating enzyme (E1), a ubiquitin‐conjugating enzyme (E2) and a ubiquitin ligase (E3) [16]. Ubiquitin‐conjugating enzyme E2O (UBE2O) is a hybrid enzyme with both E2 and E3 activities that participates in pathophysiological processes such as the nuclear transport of intracellular proteins, erythropoiesis and terminal differentiation, glycolysis, lipid metabolism and tumorigenesis [17, 18, 19, 20]. A recent study demonstrated that UBE2O facilitates extracellular matrix adhesion by selectively ubiquitinating cytosolic CTNNA1 [21]. Furthermore, UBE2O has been identified as a key regulator of HBV virion secretion [22]. Our previous research indicated that UBE2O contributes to the ubiquitination of Mxi1, thereby driving carcinogenesis and radioresistance in lung cancer [23]. However, the direct role of UBE2O in immune evasion in lung cancer and its potential impact on the efficacy of radioimmunotherapy have yet to be elucidated.

Precise regulation of the cell cycle is essential for maintaining genomic stability and normal cellular function [24]. Cyclin‐dependent kinase‐like 1 (CDKL1) shares partial sequence homology with members of the cyclin‐dependent kinase (CDK) family and is expressed predominantly in the brain, lung, kidney, and ovary, where it is involved in regulating physiological processes such as cilia formation and brain development [25, 26]. Recent studies have revealed a correlation between CDKL1 variants and an increased incidence of thoracic aortic aneurysm and dissection [27]. Furthermore, the aberrant expression of CDKL1 is significantly correlated with tumor proliferation [28, 29]. Our previous research demonstrated that CDKL1 inhibits the expression of PD‐L1 through its interaction with the transcription factor YBX1, which enhances radiosensitivity and the antitumor immune response in lung cancer [29]. However, the upstream regulatory mechanisms that govern CDKL1 remain unclear.

In this study, we identified UBE2O as a novel upstream negative regulator of CDKL1 in lung cancer. Targeting the UBE2O/CDKL1 axis has been shown to enhance antitumor efficacy by downregulating PD‐L1 expression and subsequently facilitating the proliferation and activation of CD8^+^ T cells. More importantly, genetic or pharmacological inhibition of UBE2O combined with radiotherapy and anti‐PD‐L1 immunotherapy resulted in superior antitumor efficacy, suggesting that UBE2O is a promising target for lung cancer treatment.

## 2 Results

### 2.1 UBE2O Interacts With CDKL1 In Vitro and In Vivo

**FIGURE 1:**
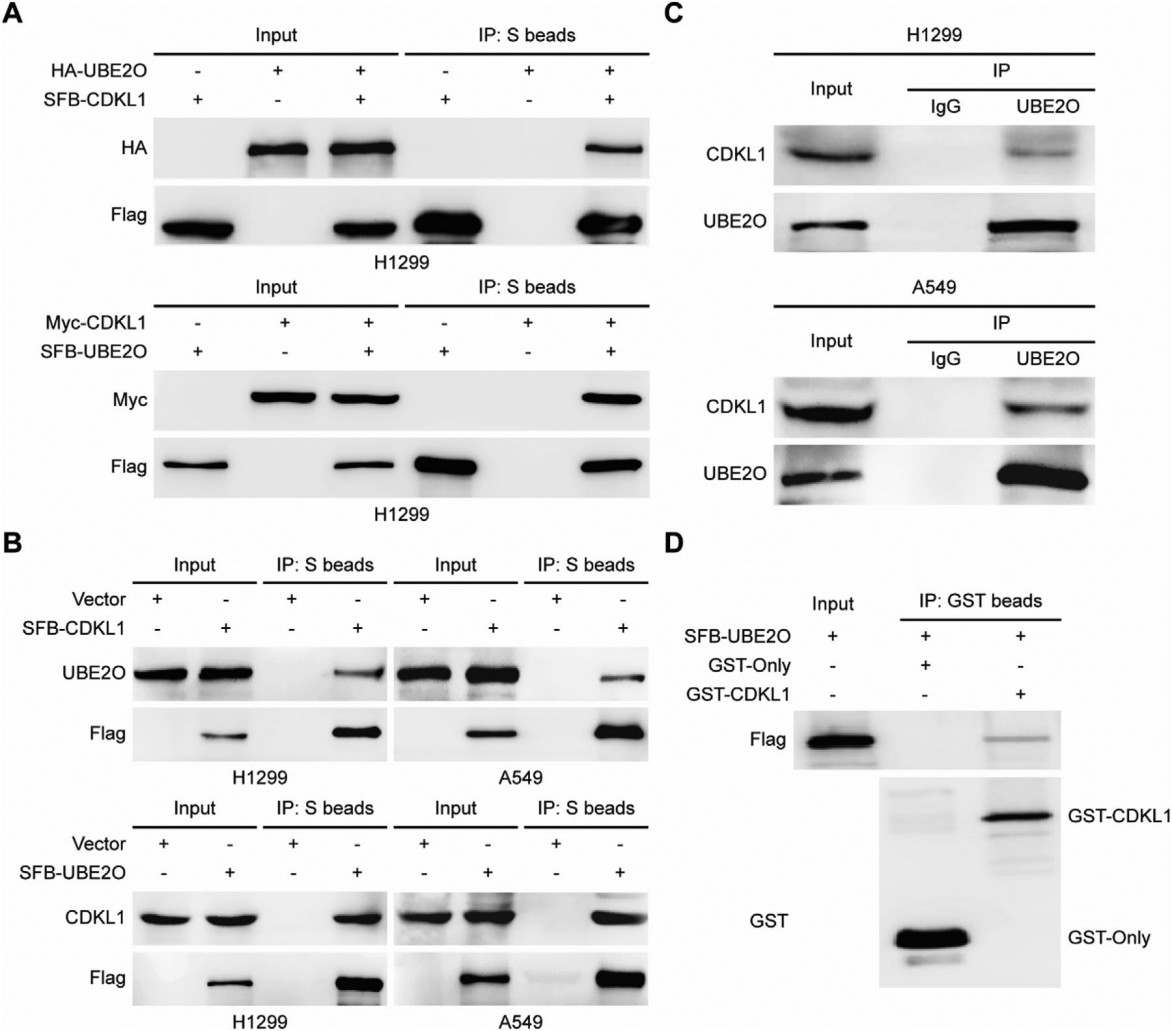
UBE2O interacts with CDKL1 in vitro and in vivo. (A) Interaction between exogenously expressed UBE2O and CDKL1 was detected in H1299 cells (*n* = 3). (B) H1299 and A549 cells were transfected with either SFB‐CDKL1 or SFB‐UBE2O for 24 h, followed by a 4‐hour treatment with MG132. Cells were collected for co‐IP with S beads (*n* = 3). (C) An endogenous interaction between UBE2O and CDKL1 was detected in both H1299 and A549 cells (*n* = 3). (D) HEK293T cells transfected with SFB‐UBE2O were lysed and incubated with the indicated proteins, followed by incubation with GST beads and subsequent detection by immunoblotting (*n* = 3).

Our previous research demonstrated that CDKL1 inhibits YBX1‐mediated PD‐L1 expression, thereby increasing radioimmunotherapy efficacy in lung cancer [29]. To investigate the upstream regulatory mechanisms of CDKL1, we identified UBE2O as a potential interacting protein through tandem affinity purification mass spectrometry [29]. We validated these mass spectrometry results using the following coimmunoprecipitation (Co‐IP) experiments. As illustrated in Figure 1A, an interaction between exogenously expressed UBE2O and CDKL1 was observed in the H1299 cell line. In both H1299 and A549 cells, ectopically expressed CDKL1 bound to endogenously expressed UBE2O, and vice versa (Figure 1B). Furthermore, we found that endogenous UBE2O bound to endogenous CDKL1 in lung cancer cells (Figure 1C). Importantly, a direct interaction between UBE2O and CDKL1 was confirmed using a GST pull‐down assay (Figure 1D). Collectively, our findings indicate that UBE2O and CDKL1 physically interact both in vitro and in vivo.

### 2.2 UBE2O Facilitates the Polyubiquitylation and Subsequent Degradation of CDKL1 in Lung Cancer

**FIGURE 2:**
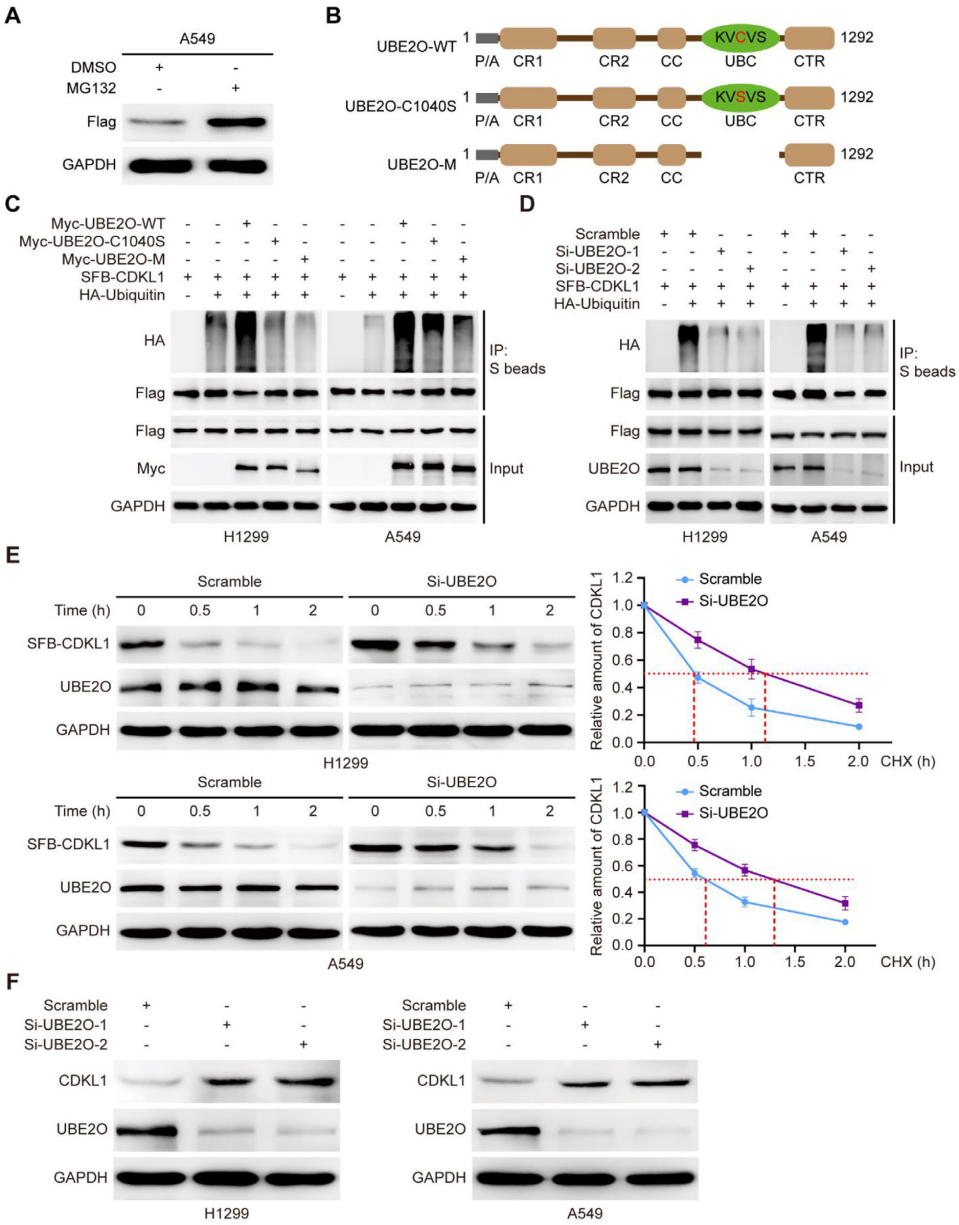
UBE2O promotes the polyubiquitylation and degradation of CDKL1 in lung cancer cells. (A) A549 cells were transfected with the SFB‐CDKL1 plasmid for 24 h. After treatment with the proteasome inhibitor MG132 for 4 h, proteins were extracted, and CDKL1 expression was assessed (*n* = 3). (B) Schematic representation of the structural domains of both the wild‐type and mutant UBE2O plasmids. (C) H1299 and A549 cells were transfected with the specified plasmids and subsequently treated with MG132 for 4 h prior to collection. The cell lysates were incubated with S beads overnight, after which the ubiquitination level of CDKL1 was evaluated (*n* = 3). (D) H1299 and A549 cells were co‐transfected with the specified siRNAs and plasmids and treated with MG132 for 4 h before being harvested, after which the cell lysates were subjected to immunoblotting (*n* = 3). (E) Following the transfection of H1299 and A549 cells with the specified siRNAs for 48 h, the cells were treated with cycloheximide (CHX, 20 µg/mL). CDKL1 protein expression was then examined by Western blotting, and the band intensities were statistically analyzed (*n* = 3). (F) H1299 and A549 cells were transfected with the indicated siRNAs for 48 h, after which Western blotting analysis was performed to determine the CDKL1 protein level (*n* = 3).

Given that the E2/E3 hybrid ubiquitin enzyme UBE2O interacts with CDKL1, we hypothesize that CDKL1 may serve as a novel ubiquitinated substrate for UBE2O. As expected, treatment with the proteasome inhibitor MG132 led to a significant increase in CDKL1 protein levels (Figure 2A), suggesting that the CDKL1 protein is unstable and subject to ubiquitination. To experimentally validate this hypothesis, we constructed two mutant plasmids and observed that wild‐type UBE2O significantly enhanced the polyubiquitylation of CDKL1 compared with both the UBE2O catalytically inactive mutant (UBE2O‐C1040S) and the mutant lacking the UBC domain (UBE2O‐M) (Figure 2B,C). In alignment with these findings, silencing UBE2O resulted in reduced CDKL1 polyubiquitylation (Figure 2D). Furthermore, protein stability assays revealed that compared with the control, the depletion of UBE2O prolonged the half‐life of the CDKL1 protein in H1299 and A549 cells (Figure 2E). In support of this observation, UBE2O knockdown led to an increase in CDKL1 protein levels in both H1299 and A549 cells (Figure 2F). Collectively, these results demonstrate that UBE2O destabilizes CDKL1 by facilitating its polyubiquitylation and subsequent degradation, indicating that CDKL1 is a bona fide substrate of UBE2O in lung cancer cells.

### 2.3 UBE2O Mediates the Ubiquitination of CDKL1 at Lysine Residue 21 (K21) in Lung Cancer

**FIGURE 3:**
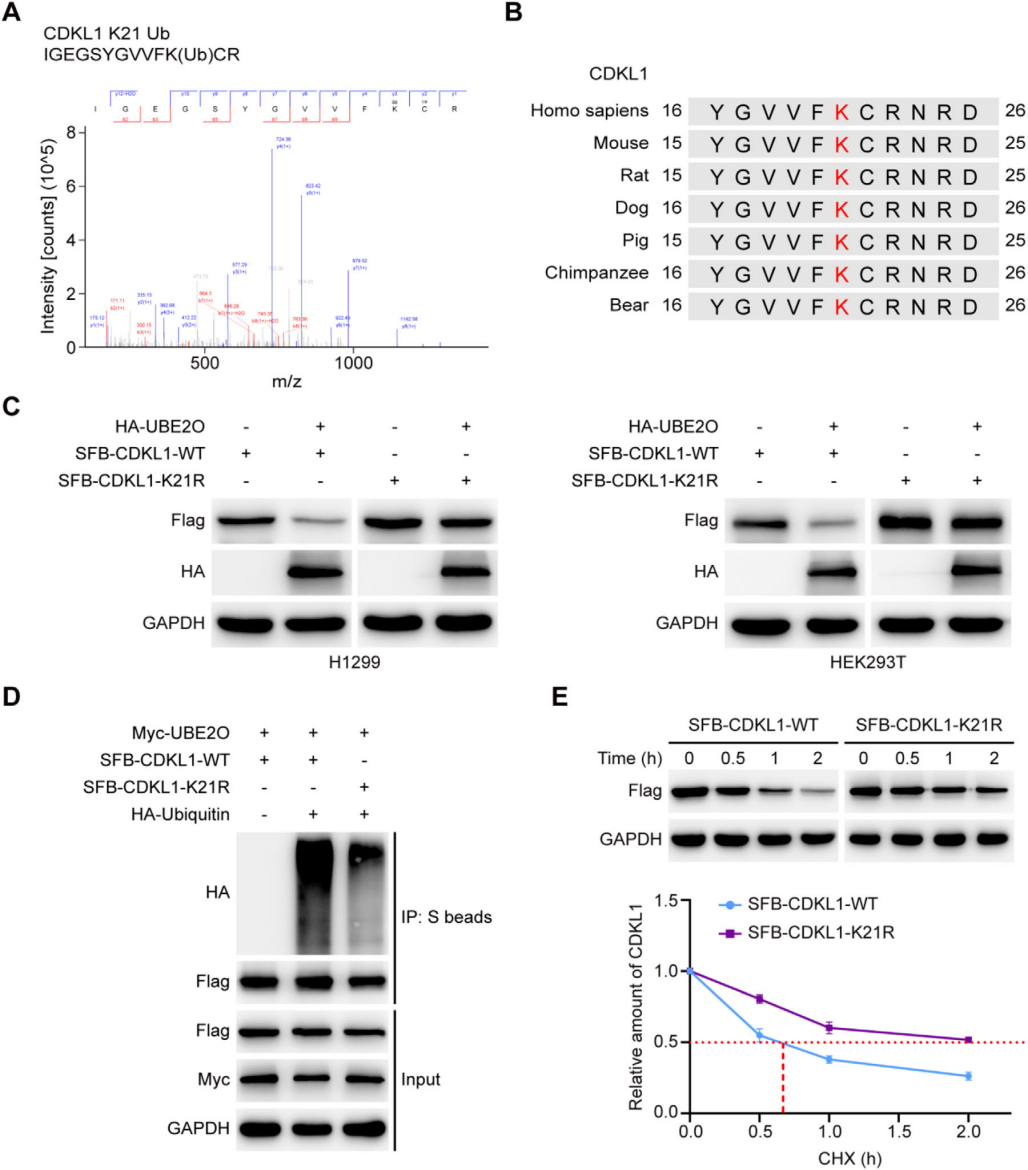
UBE2O targets CDKL1 for ubiquitination and degradation at lysine 21 (K21) in lung cancer cells. (A) Identification of the ubiquitination site on CDKL1 by mass spectrometry. (B) Comparative alignment of the amino acid sequences corresponding to K21 in CDKL1 across various species. (C) H1299 and HEK293T cells were co‐transfected with UBE2O and either wild‐type or K21R mutant CDKL1 to assess protein levels (*n* = 3). (D) H1299 cells were treated with MG132, and CDKL1 ubiquitination was evaluated by western blotting (*n* = 3). (E) Determination of the half‐life of wild‐type and K21R mutant CDKL1 in H1299 cells using cycloheximide, with protein levels quantified from three independent experiments (*n* = 3).

To identify the specific sites on CDKL1 that are ubiquitinated by UBE2O, we utilized mass spectrometry to examine all potential lysine ubiquitination sites within CDKL1 using HEK293T cells with UBE2O overexpression. As shown in Figure 3A, lysine 21 (K21) emerged as a potential ubiquitination site under conditions of UBE2O overexpression. Notably, the K21 site of CDKL1 is highly conserved across various species (Figure 3B). To validate the mass spectrometry findings, we established a CDKL1 mutant in which the lysine 21 residue was replaced with an arginine (K21R). In cells with UBE2O overexpression, the protein levels of the CDKL1‐K21R mutant remained stable, whereas those of the wild‐type CDKL1 were reduced (Figure 3C). Moreover, ubiquitination of the CDKL1 mutant was dramatically decreased by ectopic expression of UBE2O (Figure 3D). Consistent with these observations, the half‐life of the CDKL1 mutant was extended relative to that of wild‐type CDKL1 (Figure 3E). These findings indicate that the K21 residue of CDKL1 is crucial for the ubiquitination by UBE2O in lung cancer.

### 2.4 The Regulation of PD‐L1 by UBE2O Is Partially Mediated Through CDKL1 in Lung Cancer Cells

**FIGURE 4:**
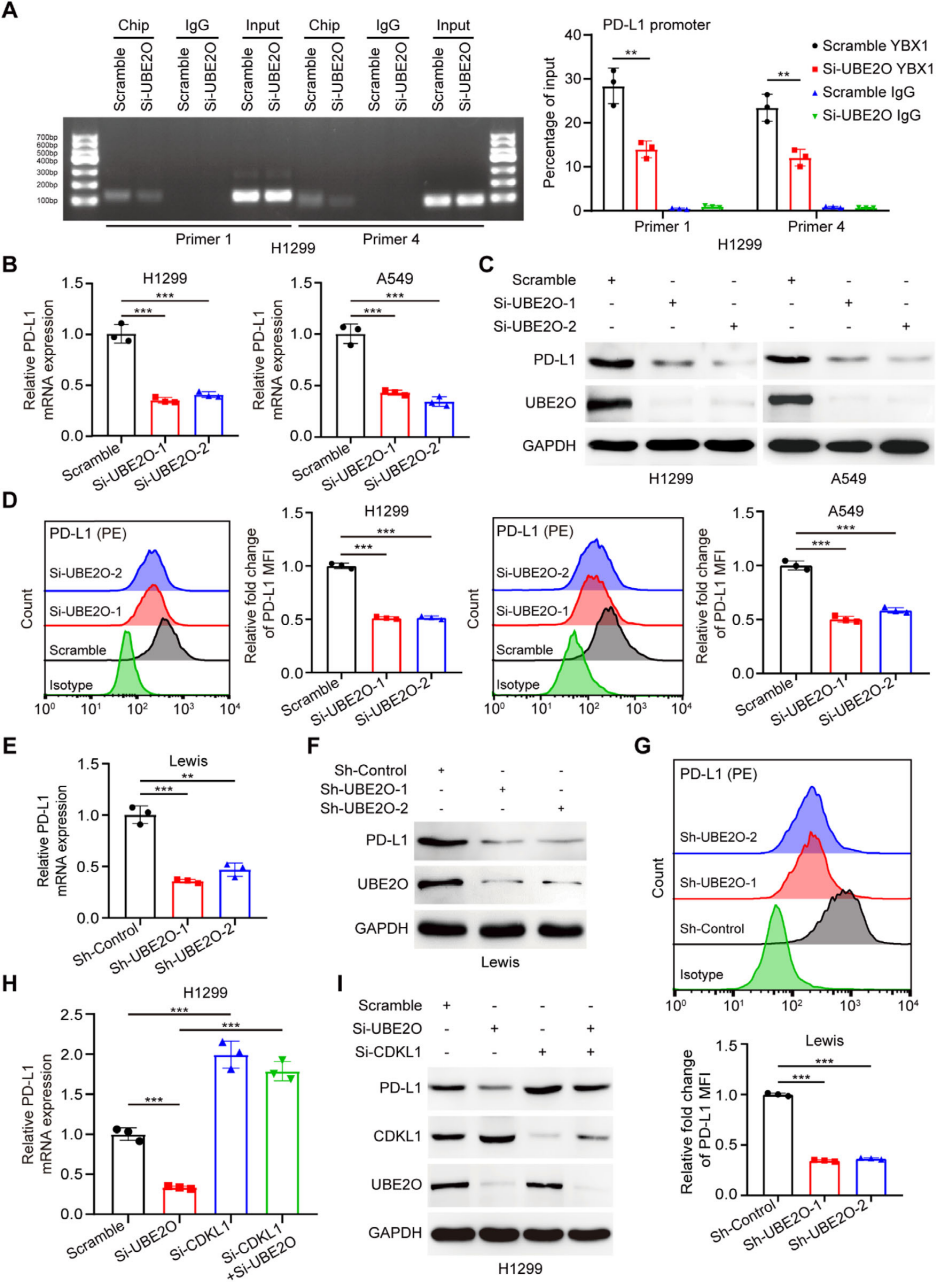
UBE2O modulates PD‐L1 expression partially through CDKL1 in lung cancer cells. (A) The left panel presents a DNA gel electropherogram from a ChIP assay conducted in H1299 cells, while the right panel provides a statistical analysis of YBX1 enrichment in the PD‐L1 promoter region (*n* = 3). (B) The mRNA levels of PD‐L1 were quantified following UBE2O knockdown (*n* = 3). (C) H1299 and A549 cells were transfected with specific siRNAs, and PD‐L1 protein expression was subsequently analyzed via Western blotting (*n* = 3). (D) The surface expression of PD‐L1 was evaluated in living H1299 and A549 cells after UBE2O silencing (*n* = 3). (E) PD‐L1 mRNA expression was assessed following stable UBE2O knockdown in Lewis cells (*n* = 3). (F) The protein levels of PD‐L1 were determined after stable UBE2O knockdown in Lewis cells (*n* = 3). (G) Upper panel: The membrane surface expression of PD‐L1 in Lewis cells was measured following the stable silencing of UBE2O. Lower panel: The quantification results of PD‐L1 expression are presented (*n* = 3). (H,I) Comparative analyses were conducted to evaluate the mRNA and protein expression levels of PD‐L1 in H1299 cells across various experimental groups (*n* = 3).

Our previous research revealed that CDKL1 suppresses PD‐L1 expression through its interaction with the transcription factor YBX1, thereby inhibiting immune evasion [29]. To explore whether UBE2O, an upstream regulator of CDKL1, also modulates PD‐L1 expression, we conducted chromatin immunoprecipitation followed by quantitative PCR (ChIP‐qPCR) and found that silencing UBE2O reduced the enrichment of YBX1 at the promoter region of PD‐L1 (Figure 4A and Figure S1). Moreover, UBE2O knockdown resulted in a reduction in PD‐L1 expression at both the transcriptional and translational levels in human lung cancer cells (Figure 4B,C). Flow cytometric analysis further confirmed that UBE2O depletion significantly decreased PD‐L1 expression on the cell membrane surface (Figure 4D). Additionally, UBE2O positively regulated PD‐L1 expression in mouse Lewis lung carcinoma cells (Figure 4E–G). Rescue experiments demonstrated that UBE2O depletion alone reduced PD‐L1 expression, whereas CDKL1 knockdown led to PD‐L1 upregulation. Notably, silencing of CDKL1 partially reversed the downregulation of PD‐L1 expression induced by UBE2O inhibition (Figure 4H,I), indicating that the modulation of PD‐L1 expression mediated by UBE2O in lung cancer cells is partially dependent on CDKL1.

### 2.5 Targeting UBE2O Activates CD8^+^ T Cells and Enhances the Antitumor Immune Response in Lung Cancer

**FIGURE 5:**
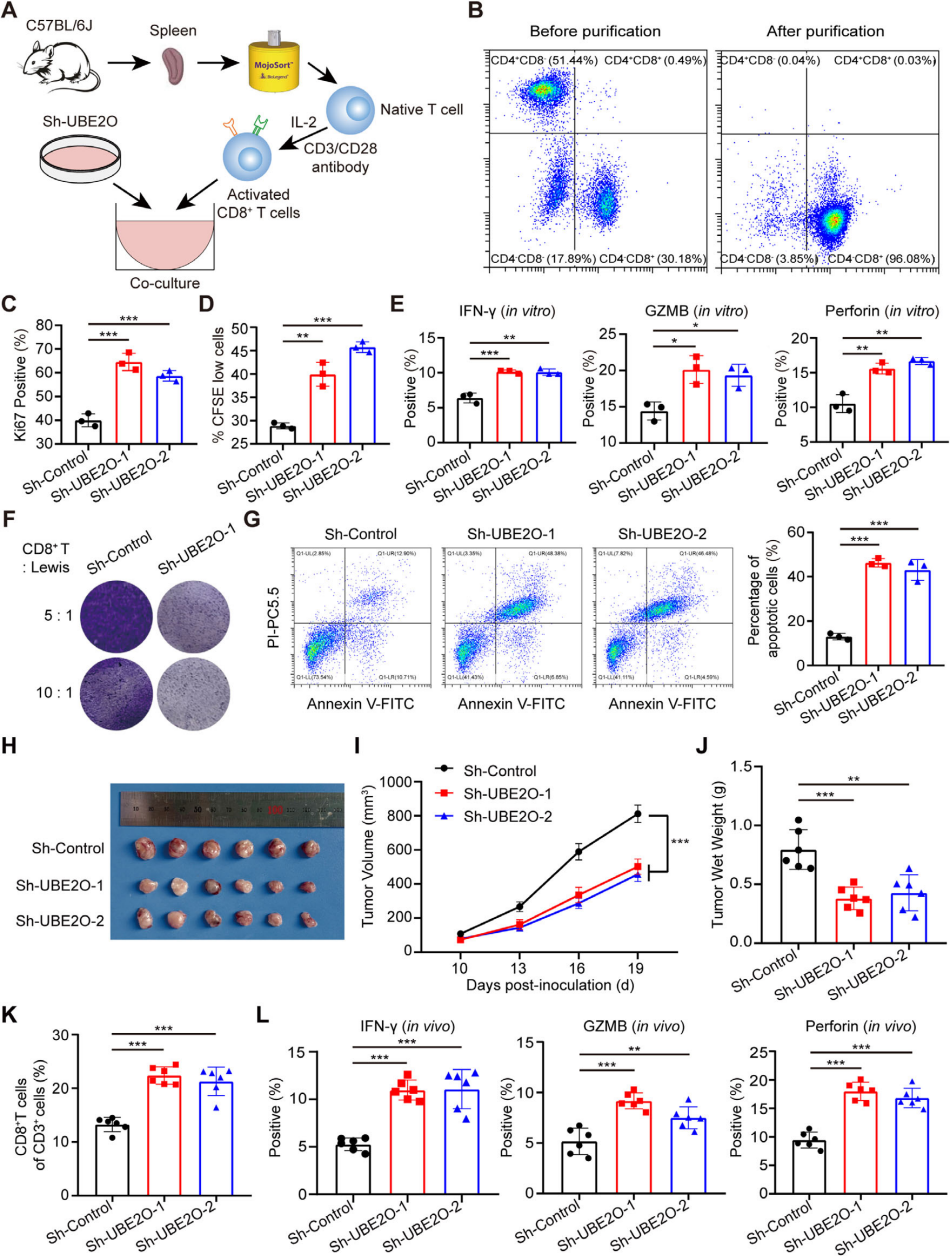
Targeting UBE2O activates CD8^+^ T cells and increases antitumor immunity. (A) Schematic representation of the constructed in vitro co‐culture model. (B) The purification efficiency of CD8^+^ T cells was validated via flow cytometry. (C) Flow cytometry was used to assess the expression level of Ki67 in CD8^+^ T cells (*n* = 3). (D) A CFSE staining assay was conducted to determine the proportion of CD8^+^ T cells with low CFSE expression levels (*n* = 3). (E) Flow cytometry was used to measure the expression levels of intracellular IFN‐γ, GZMB, and Perforin in CD8^+^ T cells in the in vitro co‐culture model (*n* = 3). (F) Lewis cells targeting UBE2O were co‐cultured with activated CD8^+^ T cells at a specific ratio for 24 h, after which the viability of the remaining tumor cells was assessed using crystal violet staining (*n* = 3). (G) Following a 24‐hour co‐culture of Lewis cells with CD8^+^ T cells, staining for apoptosis markers was performed, and the proportion of apoptotic cells was determined using flow cytometry (*n* = 3). (H) Images depicting the subcutaneous syngeneic murine model in C57BL/6J mice (*n* = 6/group). (I) Growth curves for each experimental group of mice (*n* = 6/group, mean ± SEM). (J) Tumor wet weights across different groups (*n* = 6/group). (K) The ratio of CD8^+^ T cells to CD3^+^ T cells within the tumors was evaluated on day 19 postinoculation (*n* = 6/group). (L) The intracellular expression levels of IFN‐γ, GZMB and Perforin in CD8^+^ T cells were quantified in tumors on day 19 post‐inoculation (*n* = 6/group).

PD‐L1 is widely recognized as a regulatory element that inhibits CD8^+^ T cell activity and plays a critical role in antitumor immune responses [30, 31]. Given that UBE2O upregulates the expression of PD‐L1, we hypothesized that UBE2O may influence the function of CD8^+^ T cells. To this end, we first established an in vitro co‐culture model in which the proportion of purified CD8^+^ T cells was 96.08%, as determined by flow cytometry (Figure 5A,B). Functional assays demonstrated that UBE2O silencing significantly increased the expression of Ki67 in CD8^+^ T cells and the proportion of CD8^+^ T cells with low CFSE expression (Figure 5C,D). Moreover, UBE2O knockdown led to upregulation of the expression of cytotoxic molecules, including interferon‐γ (IFN‐γ), granzyme B (GZMB) and Perforin, which are secreted by CD8^+^ T cells (Figure 5E). Subsequent cytotoxicity assays revealed that CD8^+^ T cells cultured in conditioned medium from cells in which UBE2O was silenced exhibited increased tumor cell killing ability, resulting in fewer surviving tumor cells (Figure 5F). The results of apoptosis assays further demonstrated that knockdown of UBE2O expression significantly increased tumor cell apoptosis (Figure 5G). These findings demonstrate that inhibiting UBE2O can promote the proliferation and activation of CD8^+^ T cells. Furthermore, an in vivo subcutaneous syngeneic murine model demonstrated that UBE2O knockdown led to reductions in the tumor growth rate and tumor weight (Figure 5H–J). Furthermore, in alignment with the in vitro experimental outcomes, the silencing of UBE2O resulted in increased CD8^+^ T cell infiltration within tumors and the upregulation of activated CD8^+^ T cell cytotoxicity markers, including IFN‐γ, GZMB and Perforin (Figure 5K,L). Collectively, these results indicate that targeting UBE2O can potentiate CD8^+^ T cell‐mediated immune responses in lung cancer.

### 2.6 The Combination of UBE2O Inhibition, Radiotherapy, and Anti‐PD‐L1 Immunotherapy Resulted in the Most Pronounced Antitumor Efficacy and was Associated With CD8^+^ T Cells in Lung Cancer

**FIGURE 6:**
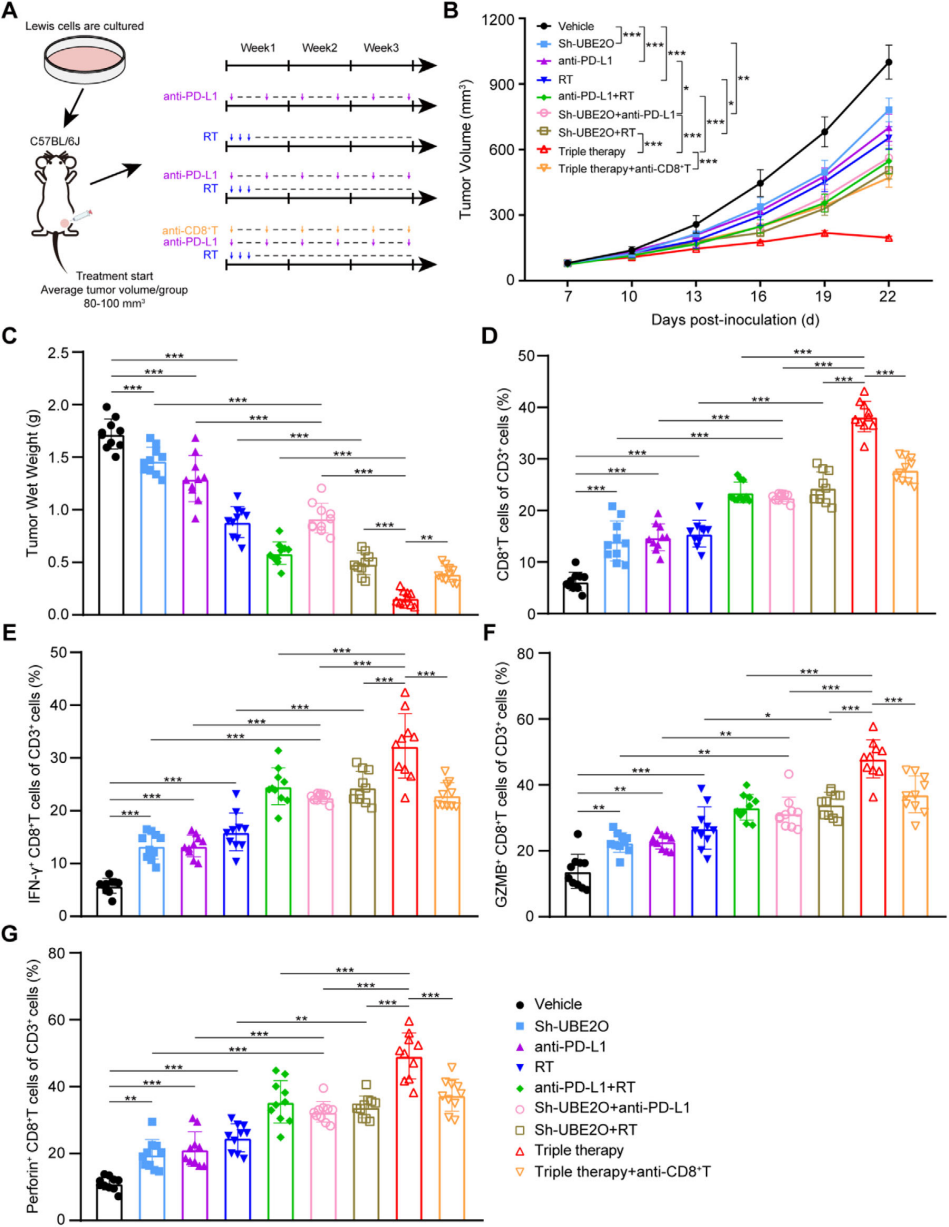
UBE2O knockdown combined with radiotherapy and anti‐PD‐L1 immunotherapy has stronger antitumor effect and was associated with CD8^+^ T cells. (A) Schematic representation of the treatment regimen for C57BL/6J mice. (B) The mice were randomly allocated to nine groups, each consisting of 10 mice, to receive distinct treatments, and tumor volumes were measured every three days (*n* = 10/group, mean ± SEM). (C) On the 22nd day post‐inoculation, the wet weight of the tumors was recorded (*n* = 10/group). (D‐G) The proportions of CD8^+^ T cells among CD3^+^ T cells and the intracellular expression levels of IFN‐γ, GZMB and Perforin in CD8^+^ T cells were measured on day 22 post‐inoculation by flow cytometry (*n* = 10/group).

Our previous research demonstrated that the inhibition of UBE2O increases radiosensitivity in lung cancer cells [23]. Considering the established immunomodulatory role of UBE2O, we hypothesize that targeting UBE2O in conjunction with radiotherapy and immunotherapy may have stronger effects against lung cancer. Indeed, experiments in murine models revealed that monotherapy, dual therapy, and triple therapy delayed tumor growth, with the triple therapy regimen having the most substantial inhibitory effect (Figure 6A–C). Flow cytometric analysis revealed increased proportions of CD8^+^ T cells within tumors across all treatment groups, with the most significant increase observed in the triple therapy group (Figure 6D). Furthermore, the three key effector molecules secreted by CD8^+^ T cells (IFN‐γ, GZMB and Perforin) were activated, and triple therapy exhibited the most robust activation (Figure 6E–G). Upon depletion of CD8^+^ T cells, the antitumor efficacy of the triple therapy was markedly diminished, confirming that the antitumor immune response elicited by the triple regimen is partially dependent on CD8^+^ T cells.

### 2.7 The Inhibition of UBE2O Activity by Arsenic Trioxide (ATO) Activates the Antitumor Immune Response in Lung Cancer

**FIGURE 7:**
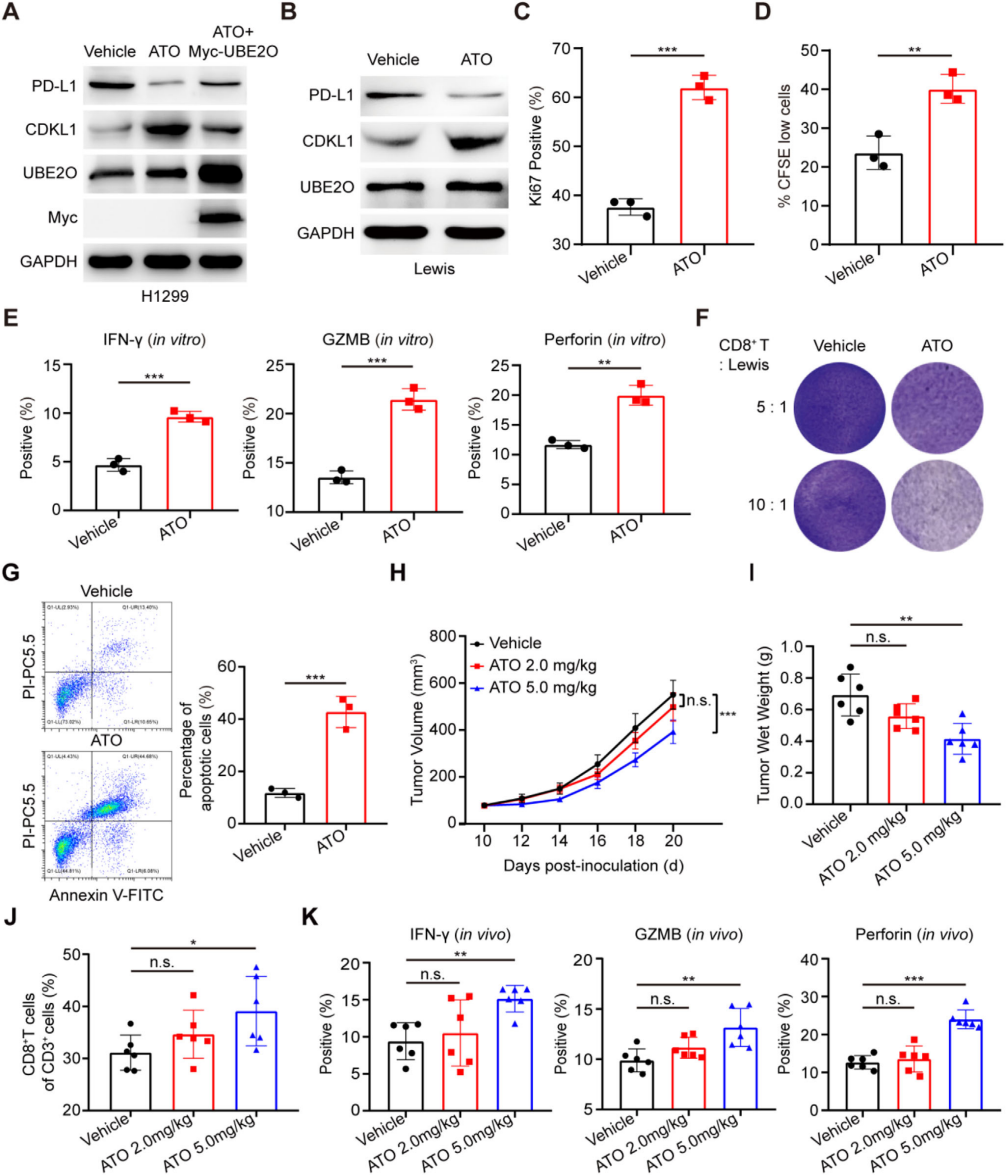
The inhibition of UBE2O activity by arsenic trioxide (ATO) activates the antitumor immune response. (A) Western blot analysis was conducted to assess the protein levels of PD‐L1 and CDKL1 in H1299 cells treated with a vehicle control, ATO alone (4 µM), or a combination of ATO and Myc‐tagged UBE2O plasmid (*n* = 3). (B) Western blot analysis was used to assess the expression levels of CDKL1 and PD‐L1 in CDKL1‐overexpressing Lewis cells treated with 5 µM ATO for 24 h (*n* = 3). (C) The expression level of Ki67 in CD8^+^ T cells was evaluated (*n* = 3). (D) The percentage of CD8^+^ T cells with low CFSE fluorescence was quantified by CFSE staining (i = 3). (E) Flow cytometry was used to detect the intracellular expression levels of IFN‐γ, GZMB and Perforin in CD8^+^ T cells after ATO treatment (*n* = 3). (F) The toxicity of CD8^+^ T cells to tumor cells after ATO treatment was assessed (*n* = 3). (G) The percentage of apoptotic Lewis cells after ATO treatment was determined via flow cytometry (*n* = 3). (H) In C57BL/6J mice, tumor volume was measured following intraperitoneal injection of 2.0 mg/kg or 5.0 mg/kg ATO (*n* = 6/group, mean ± SEM). (I) On day 20 post‐inoculation, the wet weights of the tumors were recorded (*n* = 6/group). (J,K) On day 20 post‐treatment, the proportion of CD8^+^ T cells among CD3^+^ T cells and the intracellular expression levels of IFN‐γ, GZMB, and Perforin were determined by flow cytometry (*n* = 6/group).

ATO has been shown to inhibit the enzymatic activity of UBE2O by promoting the cross‐linking of adjacent cysteine residues within its catalytic domain [32]. Considering that UBE2O knockdown enhances tumor immune responses, we examined whether ATO treatment could induce a similar biological effect. As shown in Figure 7A,B and Figure S2, treatment with ATO resulted in the upregulation of CDKL1 protein expression and a reduction in PD‐L1 protein level, whereas UBE2O protein expression remained unchanged in H1299 and Lewis cells. Importantly, UBE2O re‐expression partially reversed the ATO‐induced upregulation of CDKL1 and downregulation of PD‐L1 (Figure 7A), indicating that the regulatory effects of ATO on the expression of PD‐L1 and CDKL1 proteins are partially dependent on UBE2O. An in vitro co‐culture system was used to assess CD8^+^ T cell function. Flow cytometry analysis revealed that ATO treatment significantly increased the proliferative capacity of CD8^+^ T cells (Figure 7C,D). Moreover, ATO treatment led to increased intracellular expression of GZMB, IFN‐γ and Perforin (Figure 7E). Importantly, ATO substantially enhanced the toxicity of CD8^+^ T cells to tumor cells (Figure 7F,G). Validation of these effects in vivo revealed that ATO treatment resulted in a concentration‐dependent delay in tumor growth in murine models (Figure 7H,I). Additionally, ATO treatment increased both the infiltration and activity of CD8^+^ T cells within tumors (Figure 7J,K). Collectively, these findings indicate that ATO promotes CD8^+^ T cell proliferation and activation in lung cancer, thereby enhancing antitumor immune responses.

### 2.8 ATO Combined With Radiotherapy and Anti‐PD‐L1 Immunotherapy Exhibited Stronger Efficacy Against Lung Cancer and Acceptable Short‐Term Tolerability

**FIGURE 8:**
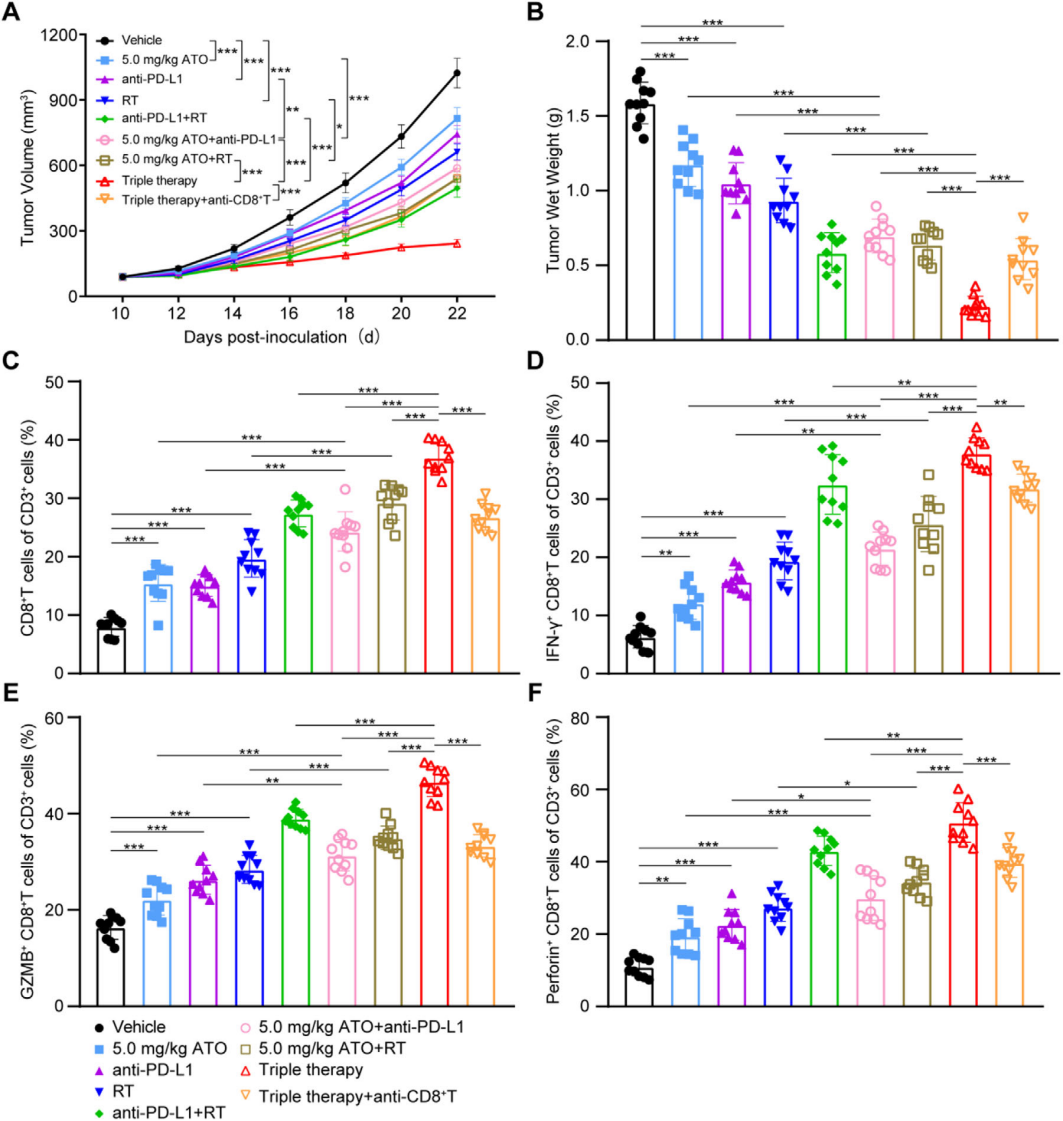
ATO combined with radiotherapy and anti‐PD‐L1 immunotherapy has a stronger antitumor effect and favorable biosafety. (A) C57BL/6J mice were randomly allocated into nine groups, each containing ten mice, to receive distinct treatments. The tumor volumes were assessed bidaily (*n* = 10/group, mean ± SEM). (B) On day 22 post‐inoculation, the wet weights of the tumors were measured (*n* = 10/group). (C–F) On day 22 post‐treatment, the proportion of CD8^+^ T cells among CD3^+^ T cells and the intracellular expression levels of IFN‐γ, GZMB and Perforin were quantified by flow cytometry (*n* = 10/group).

ATO has been demonstrated to increase the radiosensitivity of lung cancer cells [23] and facilitate the infiltration of CD8^+^ T cells. On the basis of these observations, we speculate that the integration of ATO with radiotherapy and immunotherapy could yield a stronger antitumor effect. Indeed, triple therapy markedly inhibited tumor growth (Figure 8A,B). Flow cytometry analysis revealed that triple therapy substantially increased the proportion of CD8^+^ T cells within the tumor while also increasing the secretion of cytotoxic molecules, including GZMB, IFN‐γ and Perforin (Figure 8C–F). To evaluate the safety profile of this triple therapy, we conducted hematoxylin and eosin (HE) staining on major organs and performed serum biochemical analyses. The findings indicated that, compared with those in the untreated control group, there were no significant histopathological alterations in vital organs after triple therapy, and all hematological biochemical parameters remained within normal physiological ranges (Figure S3). Therefore, these data indicate that the triple combination of ATO, radiotherapy and anti‐PD‐L1 immunotherapy elicits robust antitumor efficacy while exhibiting acceptable short‐term tolerability in preclinical lung cancer mouse models.

## 3 Discussion

In this study, we present substantial evidence elucidating the biological function of UBE2O in conferring resistance to radioimmunotherapy in lung cancer. Mechanistically, our research identifies UBE2O as a novel upstream regulatory molecule of CDKL1 that modulates PD‐L1 expression partially through the CDKL1/YBX1 axis. Functionally, we demonstrate that targeting UBE2O increases the proliferation and activation of CD8^+^ T cells, thereby augmenting antitumor immunity. Notably, the combination of ATO, a cross‐linking inhibitor of UBE2O, with radiotherapy and anti‐PD‐L1 immunotherapy achieves optimal antitumor immune efficacy while maintaining an acceptable short‐term tolerability.

Protein ubiquitination represents a critical posttranslational modification that is involved in nearly all cellular processes within eukaryotes. Disruption of the ubiquitination pathway can lead to the development of various diseases, including autoimmune disorders, metabolic syndrome and cancer [13, 33]. In this study, through proteomics technology, we identified UBE2O as a novel binding protein of CDKL1. The interaction between CDKL1 and UBE2O was subsequently validated both in vivo and in vitro. Additionally, we demonstrated that the enzymatic activity of UBE2O influences the ubiquitination of CDKL1. UBE2O binds to CDKL1 and facilitates its polyubiquitination and subsequent proteasomal degradation at the K21 residue, thereby negatively regulating its protein stability. These findings provide the first evidence that UBE2O acts as a novel ubiquitin enzyme for CDKL1, mediating its degradation via the UPS in lung cancer. Furthermore, our previous study has demonstrated that UBE2O is overexpressed and predicts poor prognosis in lung cancer patients, highlighting the need for clinical validation of the UBE2O/CDKL1 axis in future research.

PD‐1/PD‐L1 represents a critical immune checkpoint that plays a pivotal role in transmitting immunosuppressive signals and attenuating the cytotoxic T cell‐mediated immune response [34, 35]. PD‐L1 is subject to various posttranslational modifications, such as ubiquitination, lactylation, and palmitoylation [35, 36, 37]. Previous studies have shown that YBX1 can bind to the PD‐L1 promoter and induce its expression, consequently suppressing the activation of CD8^+^ T cells [38]. Our previous investigations revealed that CDKL1 suppresses PD‐L1 expression by reducing YBX1 binding to the PD‐L1 promoter [29]. In this study, we elucidated that UBE2O modulates PD‐L1 expression partially through CDKL1 in lung cancer. Moreover, targeting UBE2O increases the proliferation and activation of CD8^+^ T cells, thereby augmenting antitumor immune responses. Experimental results with animal models demonstrate that the combination of UBE2O inhibition with radiotherapy and anti‐PD‐L1 immunotherapy produces the most potent antitumor effects. Consequently, our study not only clarifies the molecular mechanisms by which UBE2O regulates PD‐L1 expression but also reveals novel biological roles of UBE2O in conferring resistance to radioimmunotherapy in lung cancer. However, our study could not rule out the possibility that UBE2O may affect PD‐L1 through additional substrates or pathways, and this warrants further investigation.

ATO has demonstrated considerable clinical efficacy in the treatment of hematological malignancies, with a pronounced effect in cases of acute promyelocytic leukemia (APL) [39]. Recent evidence suggests that ATO exerts its antitumor effect by inhibiting the Hedgehog/GLI signaling pathway [40]. Additionally, ATO can induce necroptosis and ferroptosis via oxidative stress pathways, which in turn stimulates tumor‐specific immune responses [41]. In the present study, we demonstrated that ATO can upregulate the expression of CDKL1 and downregulate the expression of PD‐L1 in lung cancer models, and this effect is partially dependent on UBE2O. Furthermore, targeting UBE2O via ATO increases CD8^+^ T cell proliferation and activation while effectively inhibiting tumor growth in a subcutaneous syngeneic murine model. Notably, the combination of ATO, radiotherapy and anti‐PD‐L1 immunotherapy resulted in the most pronounced sensitization to radioimmunotherapy while maintaining an acceptable short‐term tolerability. Given that ATO is a pleiotropic agent rather than a specific UBE2O inhibitor, we speculate that its role in enhancing the antitumor immune response likely involves additional mechanisms, including direct binding to other proteins such as AMPK and broader cytotoxic/immunogenic effects. Nevertheless, our study elucidates a novel regulatory mechanism by which ATO modulates radioimmunotherapy sensitization in lung cancer, highlighting its potential value for clinical translation.

**FIGURE 9:**
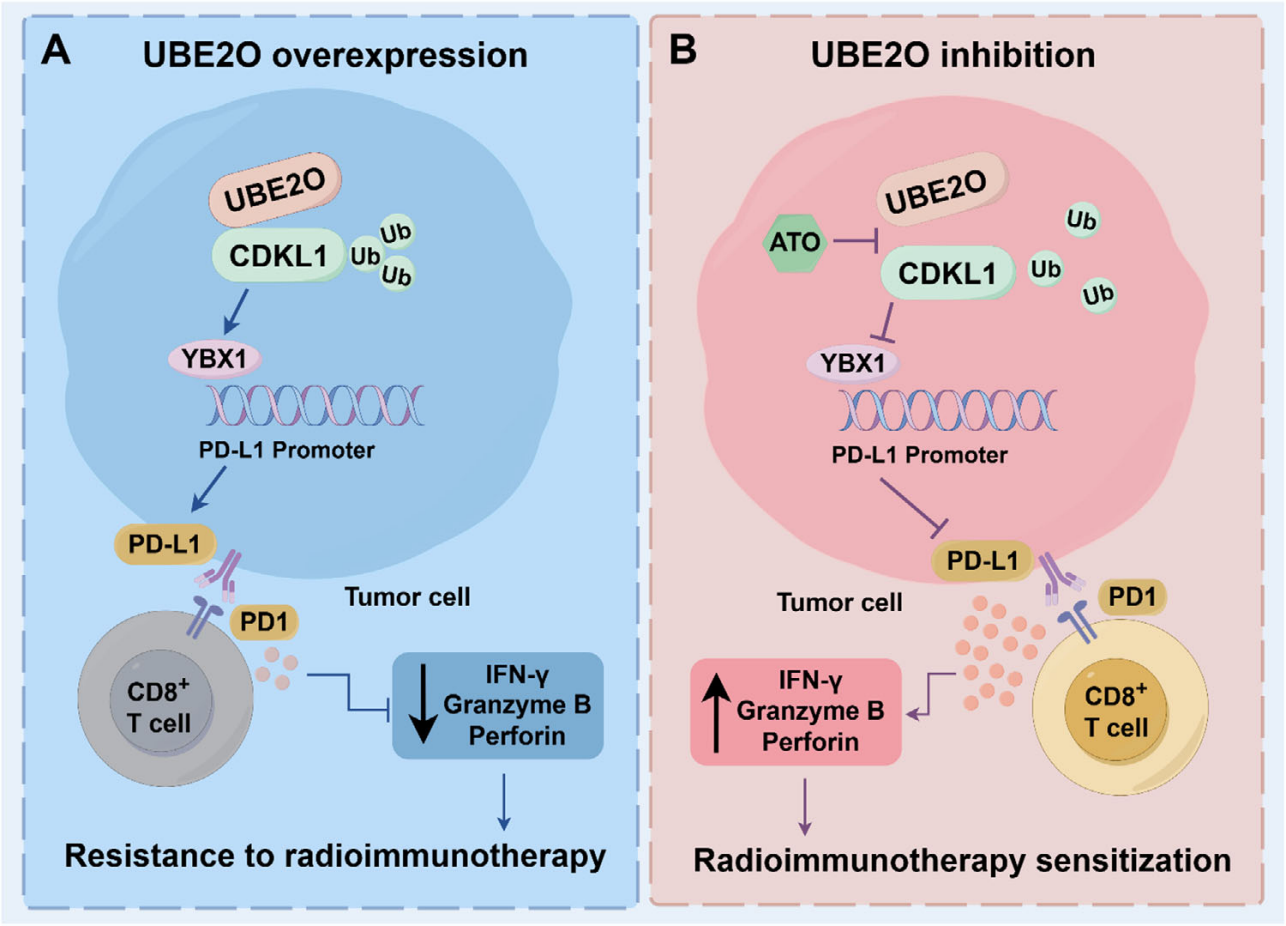
Schematic diagram illustrating the mechanism by which UBE2O modulates resistance to radioimmunotherapy in lung cancer. (A) The E2/E3 hybrid ubiquitin enzyme UBE2O interacts with CDKL1 to facilitate its ubiquitination and subsequent degradation. This process diminishes the binding of CDKL1 to the transcription factor YBX1, thereby increasing the YBX1‐mediated transcription of the downstream target gene PD‐L1. Consequently, this cascade inhibits CD8^+^ T cell activity, ultimately conferring resistance to radioimmunotherapy. (B) The inhibition of UBE2O reduces the ubiquitination and degradation of CDKL1, which enhances the binding of CDKL1 to YBX1. This interaction suppresses the transcription of the downstream target gene PD‐L1, thereby increasing CD8^+^ T cell activity and the immune response, ultimately leading to sensitization to radioimmunotherapy.

In summary, this study is the first to elucidate a novel mechanism by which UBE2O regulates the degradation of CDKL1 through direct interaction and ubiquitination. Our findings indicate that UBE2O positively modulates PD‐L1 expression, thereby inhibiting CD8^+^ T cell activity and driving immune evasion. Moreover, the combination treatment strategy involving UBE2O targeting, radiotherapy and anti‐PD‐L1 immunotherapy demonstrated the robust antitumor efficacy while maintaining an acceptable short‐term tolerability (Figure 9A,B). This approach presents a promising therapeutic intervention for sensitizing lung cancer to radioimmunotherapy.

## 4 Experimental Section

### 4.1 Cell Culture and Transfection

Two human lung cancer cell lines, H1299 (RRID: CVCL_0060) and A549 (RRID: CVCL_0023), along with HEK293T (RRID: CVCL_0063) and Lewis cells (RRID: CVCL_4358), were purchased from the American Type Culture Collection. All culture media were supplemented with a penicillin‐streptomycin‐amphotericin B solution (Beyotime). All the cell lines were tested for mycoplasma contamination and authenticated using short tandem repeat (STR) profiling. The siRNAs were transfected using Lipofectamine RNAiMAX (Invitrogen) for 48 h. Plasmid transfection was performed with Lipofectamine 2000 (Invitrogen) for 24 h. The siRNA sequences used in this study were as follows:

si‐UBE2O‐1: 5′‐GGUUGUAGAGUUGAAAGUUTT‐3′;
si‐UBE2O‐2: 5′‐CCACCCAGUGUGAAACCAATT‐3′ [23]; and
si‐CDKL1: 5′‐GCAAGUGUUUAGCACGAAU‐3′ [29].

### 4.2 Antibodies and Reagents

The anti‐UBE2O antibody (A301‐873A, RRID: AB_1309799) was obtained from Bethyl Laboratories, and the anti‐CDKL1 antibody (AB136129, RRID: AB_3741640) was purchased from Abcam. Anti‐YBX1 (20339‐1‐AP, RRID: AB_10665424), anti‐GAPDH (60004‐1‐Ig, RRID: AB_2107436), anti‐PD‐L1 (17952‐1‐AP, RRID: AB_10597552) and anti‐Myc (66004‐1‐Ig, RRID: AB_2881489) antibodies were purchased from Proteintech. An anti‐HA antibody (3724S, RRID: AB_1549585) was purchased from Cell Signaling Technology. Anti‐Flag (AE005, RRID: AB_2770401) and anti‐GST (AE006, RRID: AB_2771923) antibodies were obtained from ABclonal. To analyze the tumor mouse model, anti‐mouse PD‐L1 (BioXCell, BE0101, RRID: AB_10949073) and anti‐mouse CD8a (BioXCell, BE0061, RRID: AB_1125541) antibodies were used. The following antibodies and reagents purchased from BioLegend were used for flow cytometry analysis: anti‐CD16/32 (101320, RRID: AB_1574975), Zombie Aqua Fixable Viability Kit (423101, RRID: AB_3741647), anti‐CD45 (103154, RRID: AB_2572116), anti‐CD3 (100336, RRID: AB_11203705), anti‐CD8a (100706, RRID: AB_312745), anti‐CD4 (100525, RRID: AB_312726), anti‐IFN‐γ (505808, RRID: AB_315402), anti‐Perforin (154406, RRID: AB_2721641), anti‐GZMB (372204, RRID: AB_2687028), anti‐Ki‐67 (652410, RRID: AB_2562141), anti‐human‐PD‐L1 (329706, RRID: AB_940368) and anti‐mouse‐PD‐L1 (124308, RRID: AB_2073556). The following reagents were also used in this study: MG132 (Millipore, 474790), arsenic trioxide (ATO) (Beijing Shuanglu Pharmaceutical Co, H20080664), CFSE (Sigma, 21888) and IL‐2 (MCE, HY‐P7077).

### 4.3 Western Blotting and Immunoprecipitation

Designated cells were lysed in NETN buffer, after which the proteins were extracted. Protein concentrations were determined using the Bradford assay. Following protein quantification, protein loading buffer was added, and the samples were heated to 100°C for 10 min. The proteins were then separated by SDS‐PAGE, transferred onto PVDF membranes, and incubated overnight with the appropriate antibodies. For immunoprecipitation experiments, the protease inhibitor MG132 was added 4 h prior to cell treatment. The supernatants were incubated overnight with S‐protein agarose (Millipore, 69704) or Protein A/G PLUS‐Agarose (Santa Cruz, sc‐2003) at 4°C, washed and subjected to immunoblotting.

### 4.4 GST Pull‐Down Assay

The GST‐vector and GST‐CDKL1 plasmids were introduced into BL21 cells and subsequently purified. The SFB‐UBE2O plasmid was transfected into HEK293T cells. After 24 h, the cell lysates were incubated with the purified GST‐Only and GST‐CDKL1 fusion proteins. Afterward, activated GST beads were added to the mixture for incubation overnight at 4°C. The beads were then washed five times with NETN buffer the following day, and the proteins were verified by Western blotting.

### 4.5 Ubiquitination Assay

Cells were first transfected with the corresponding siRNAs, followed by transfection with SFB‐tagged and ubiquitin plasmids the next day. After a 24‐hour incubation period, the cells were treated with 10 µM MG132 for 4 h, after which protein extraction was performed. The cell lysates were incubated with S‐protein agarose overnight at 4°C. The samples were subsequently subjected to five washes with NETN buffer before being analyzed via Western blotting.

### 4.6 Protein Half‐Life Assessment

Specific siRNAs and plasmids were transfected into cells cultured in six‐well plates. Cycloheximide (CHX; Sigma, 20 µg/mL) was added to the wells 2, 1, or 0.5 h before protein extraction. Western blot analysis was conducted, and densitometric analysis of each band was performed using ImageJ software.

### 4.7 Mass Spectrometric Identification of Ubiquitination Sites on CDKL1

To systematically delineate ubiquitination sites on CDKL1, HEK293T cells were transiently co‐transfected with plasmids encoding SFB‐CDKL1, Myc‐UBE2O and HA‐ubiquitin. Twenty‐four hours post‐transfection, the cells were treated with the proteasome inhibitor MG132 for 4 h to promote the accumulation of ubiquitinated proteins prior to the preparation of protein lysates. Immunoprecipitation was conducted under denaturing conditions, and the eluted protein complexes were analyzed using high‐resolution liquid chromatography‐tandem mass spectrometry (LC‐MS/MS).

### 4.8 Establishment of Stable Lung Cancer Cell Lines

HEK293T cells were transfected with the psPAX2, pMD2.G and sh‐UBE2O plasmids when the cell confluency reached 30% to 50%. Forty‐eight hours post‐transfection, the cell supernatants were collected and filtered. Lewis cells were subsequently infected with viral supernatants mixed at a 1:1 ratio with complete medium supplemented with 10 µg/mL polybrene for two consecutive days. Stable cell lines were then selected using puromycin at a concentration of 2 µg/mL. The shRNA sequences used were as follows:

Sh‐UBE2O‐1: 5‐GACATCAAGAAGCTACAGGAA‐3; and
Sh‐UBE2O‐2: 5‐TCGTCATCCGCATCGGCAATA‐3.

### 4.9 Quantitative Reverse Transcription‐PCR (qRT‐PCR) Assay

Total RNA was isolated from the cells and reverse transcription was subsequently performed using ABScript III RT Master Mix (ABclonal, RK20429) to generate cDNA for subsequent PCR analyses. The sequences of the primers used for quantitative real‐time PCR (qRT‐PCR) are listed in Table S1.

### 4.10 Chromatin Immunoprecipitation (ChIP)

The ChIP Assay Kit (P2078) was obtained from Beyotime and utilized following the manufacturer's protocol. First, the cells were incubated with 1% formaldehyde at 37°C for 10 min to facilitate cross‐linking. The reaction was then quenched by the addition of glycine solution (10×) and allowed to sit at room temperature for 5 min. The cell pellet was subsequently collected and lysed using SDS lysis buffer, followed by sonication and chromatin fragmentation. The processed samples were incubated overnight at 4°C with A+G agarose/salmon sperm DNA and IgG/anti‐YBX1 antibodies. The following day, the precipitates were washed and subjected to PCR amplification. The amplified products were detected via DNA gel electrophoresis. The sequences of the primers used for ChIP are listed in Table S2.

### 4.11 CCK‐8 Assay

The cell suspensions were added to 96‐well plates at an approximate density of 2 × 10^3^ cells per well. The cells were then stimulated with various concentrations of arsenic trioxide (ATO) and incubated for 24 h. Subsequently, 10 µL of CCK‐8 solution was added to each well, followed by incubation in a cell incubator at 37°C for an additional 1.5 h. The absorbance of each well at 450 nm was measured.

### 4.12 Flow Cytometry Analysis of Cell Surface PD‐L1 Expression

Cells were digested with trypsin, after which the precipitate was washed two to three times with phosphate‐buffered saline (PBS). Subsequently, the PD‐L1 flow‐through antibody was prepared in PBS and incubated on ice for 30 min in the dark. The precipitate was then washed twice with PBS and ultimately resuspended in 200 µl of PBS before being transferred to a flow‐through tube for subsequent detection.

### 4.13 Proliferation and Activity Detection of CD8^+^ T Cells

CD8^+^ T cells were isolated from the spleens of wild‐type C57BL/6J mice utilizing a MiniMACS Separator. Naive CD8^+^ T cells were subsequently stimulated and activated in RPMI 1640 medium supplemented with recombinant mouse IL‐2 (20 ng/mL, MCE, HY‐P7077), anti‐mouse CD3ε antibody (2 µg/mL, BioLegend, 100340), and anti‐mouse CD28 antibody (2 µg/mL, BioLegend, 102116). The proliferative capacity of CD8^+^ T cells was assessed through the detection of Ki‐67 expression and the execution of CFSE staining assays. The activation status of CD8^+^ T cells was determined by measuring the expression levels of intracellular effector molecules, including IFN‐γ, GZMB and Perforin. For the detection of Ki‐67, the cells were harvested and washed, followed by fixation and permeabilization. The cells were subsequently incubated with an anti‐Ki‐67 antibody at 4°C in the dark, washed, and analyzed via flow cytometry. For CFSE staining, freshly isolated CD8^+^ T cells were labeled with CFSE dye and incubated at 4°C in the dark. After quenching and washing of the labeled cells, they were co‐cultured with tumor cells for three days in the presence of IL‐2 and anti‐CD3ε/CD28 antibodies. Finally, the proportion of CFSE‐positive cells was analyzed by flow cytometry. For in vivo experiments, tumor tissues were enzymatically digested and mechanically dissociated to prepare single‐cell suspensions. Dead cells were labeled with Zombie NIR viability dye at 4°C in the dark. The cells were then stained with fluorochrome‐conjugated anti‐CD45, anti‐CD3, anti‐CD4, and anti‐CD8 antibodies at 4°C in the dark for 30 min. After being washed, the cells were fixed and permeabilized, followed by intracellular staining with anti‐IFN‐γ, anti‐GZMB and anti‐Perforin antibodies at 4°C in the dark for 30 min. After extensive washing, the frequency of CD8^+^ T cells among CD3^+^ T cells and the percentage of cells that were positive for intracellular functional molecules (IFN‐γ, GZMB and Perforin) within CD8^+^ T cells were quantified by flow cytometry to evaluate the effector function of CD8^+^ T cells.

### 4.14 In Vitro T Cell‐mediated Tumor Cell Killing Assay

Mouse splenocytes were isolated using negative selection kits, and CD8^+^ T cells were activated with anti‐CD3 and anti‐CD28 antibodies for 72 h. CD8^+^ T cells were then proportionally inoculated into 24‐well plates containing either UBE2O knockdown or control Lewis cells, followed by a 24‐hour pretreatment with 5 µM ATO. After an additional 24 h, the culture medium was removed. The cells were subsequently washed, fixed with 4% paraformaldehyde, and stained with crystal violet for 1 h.

### 4.15 Apoptosis Assessment

Activated CD8^+^ T cells were co‐cultured with Lewis cells with stable knockdown of UBE2O or with Lewis cells that had been treated with ATO for 24 h. These co‐cultures were incubated in 24‐well plates at predetermined ratios for 24 h. The cells and culture supernatants were subsequently harvested, and the cells were subjected to staining using an Annexin V‐FITC Apoptosis Detection Kit (Beyotime, C1062). Apoptotic cells were detected using a flow cytometer.

### 4.16 Animal Experiments

This study was approved by the Institutional Animal Care and Use Committee at Tongji Medical College, Huazhong University of Science and Technology (IACUC Number: 3607). Cells were prepared, resuspended in PBS, and diluted to a concentration of 1 × 10^7^ cells/mL. Female C57BL/6J mice (RRID: IMSR_JAX:000664) aged five weeks were randomly allocated to different groups. Subcutaneous tumor volume was calculated using the formula (length × width^2^)/2. The mice were subjected to treatment when the tumor volume reached 80–100 mm^3^. For radiotherapy, 8 Gy of radiation was administered over three consecutive days. The anti‐mouse PD‐L1 monoclonal antibody (10.0 mg/kg) and anti‐mouse CD8a antibody (200 µg/mouse) were injected every 4 days. ATO (5.0 mg/kg) treatment was performed daily via intraperitoneal injection as needed. To assess drug safety, key organs were subjected to hematoxylin and eosin (HE) staining to evaluate potential histomorphological alterations. Additionally, peripheral blood samples were collected from each group of mice for biochemical analysis to determine whether the biochemical parameters of critical organs remained within the normal range.

### 4.17 Statistical Analysis

CytExpert software (RRID: SCR_017217) was used for analysis of the flow cytometry data, and GraphPad Prism (RRID: SCR_002798) software was used for statistical analyses. Unless otherwise specified, all the data are presented as the mean ± standard deviation (SD) of three independent biological replicates. Statistical significance was determined using either a t test or two‐way ANOVA, and a *p* value of less than 0.05 was considered to indicate statistical significance (n.s. *p* > 0.05, * *p* < 0.05, ** *p* < 0.01, *** *p* < 0.001).

## Author Contributions

**Huichan Xue**: methodology, formal analysis, data curation, and writing – original draft. **Xiaohua Jie**: methodology, data curation, and writing – original draft. **Zhiwei Liu**: methodology and data curation. **Ruoxin Fang**: data curation. **Shuo Wu**: data curation. **Xiaorong Dong**: supervision, project administration, and investigation. **Shuangbing Xu**: conceptualization, supervision, project administration, investigation, funding acquisition, and writing – review & editing.

## Conflicts of Interest

The authors declare no conflicts of interest.

## Supporting information




**Supporting File**: advs76223‐sup‐0001‐SuppMat.docx.

## Data Availability

The data that support the findings of this study are available from the corresponding author upon reasonable request.
