# Supplementary material for: UBE2O Drives Immune Evasion and Radioimmunotherapy Resistance in Lung Cancer by Degrading CDKL1 to Induce PD‐L1 Transcription
Source: Adv Sci (Weinh). 2026 Jun 22;13(52):e76223. doi: 10.1002/advs.76223 (PMC13336739; doi:10.1002/advs.76223)
Supplement: Supplementary file 1 [file ADVS-13-e76223-s001.docx]

**FIGURE S1 |** (A) Schematic diagram illustrating the design of the primers used for ChIP-PCR. (B) The primers were verified in H1299 cells (n = 3).

**FIGURE S2 |** The half-maximal inhibitory concentration (IC_50_) of ATO in Lewis cells was determined using a CCK8 assay (n = 3).

**FIGURE S3 |** Assessment of the safety profile of the therapeutic regimen. (A) HE-stained images of key organs, including the heart, liver, spleen, lungs, and kidneys, were obtained from mice in both the control and experimental groups. Scale bar, 20 μm. (B) Peripheral blood biochemical indices of the mice were measured, and the data were standardized using Z score statistical analysis.

| **Genes** | **Sequences (5’--3’)** |
| --- | --- |
| *PD-L1* (human) | F：TCACTTGGTAATTCTGGGAGC |
|  | R：CTTTGAGTTTGTATCTTGGATGCC |
| *PD-L1* (mouse) | F：ACTTGCTCATCTTCCTTTT |
|  | R：TTTACTATCACGGCTCCA |
| *UBE2O* (human) | F：AACATCCGCTCCAACGACCTCT |
|  | R：CCAGACTGTACCACACCGTAGA |
| *UBE2O* (mouse) | F：CAGCAGCAAAGTGGAAGT |
|  | R：CAGACTCGATGTTGTATAAGTG |
| *GAPDH* (human) | F：AGAAGGCTGGGGCTCATTTG |
|  | R：AGGGGCCATCCACAGTCTTC |
| *GAPDH* (mouse) | F：TCAACGGCACAGTCAAGG |
|  | R：TTAGTGGGGTCTCGCTCC |

F, forward primer R, reverse primer.

**Table S1 |** Sequences of primers used for qRT-PCR.

| **Genes** | **Primer** | **Sequences (5’--3’)** |
| --- | --- | --- |
| *PD-L1* | Primer 1 | F：TCTGACTTCTGACTTCGTT |
|  |  | R：TCTCCAAAGTCAGCCAAT |
|  | Primer 2 | F：TGTAAACCGAGGGCATTG |
|  |  | R：TAAAGCCATTTCCAGACT |
|  | Primer 3 | F：TGCTTTGATTGTCTTCTT |
|  |  | R：ACCGGTACACTCCAGGCT |
|  | Primer 4 | F：TCCCTTACTGAGGAGAAA |
|  |  | R：GAAACACCAAAGAAACAT |

F, forward primer R, reverse primer.

**Table S2 |** Sequences of primers used for ChIP-PCR.
